# Redox and Calcium Alterations of a Müller Cell Line Exposed to Diabetic Retinopathy-Like Environment

**DOI:** 10.3389/fncel.2022.862325

**Published:** 2022-03-18

**Authors:** Clarissa Rosato, Barbara Bettegazzi, Pia Intagliata, Maria Balbontin Arenas, Daniele Zacchetti, Antonella Lanati, Gianpaolo Zerbini, Francesco Bandello, Fabio Grohovaz, Franca Codazzi

**Affiliations:** ^1^Vita-Salute San Raffaele University, Milan, Italy; ^2^Division of Neuroscience, IRCCS San Raffaele Scientific Institute, Milan, Italy; ^3^Valore Qualità, Pavia, Italy; ^4^Complications of Diabetes Unit, Diabetes Research Institute (DRI), IRCCS San Raffaele Scientific Institute, Milan, Italy; ^5^Department of Ophthalmology, IRCCS San Raffaele Scientific Institute, Milan, Italy

**Keywords:** Müller cells, inflammation, oxidative stress, calcium, diabetic retinopathy

## Abstract

Diabetic retinopathy (DR) is a common complication of diabetes mellitus and is the major cause of vision loss in the working-age population. Although DR is traditionally considered a microvascular disease, an increasing body of evidence suggests that neurodegeneration is an early event that occurs even before the manifestation of vasculopathy. Accordingly, attention should be devoted to the complex neurodegenerative process occurring in the diabetic retina, also considering possible functional alterations in non-neuronal cells, such as glial cells. In this work, we investigate functional changes in Müller cells, the most abundant glial population present within the retina, under experimental conditions that mimic those observed in DR patients. More specifically, we investigated on the Müller cell line rMC-1 the effect of high glucose, alone or associated with activation processes and oxidative stress. By fluorescence microscopy and cellular assays approaches, we studied the alteration of functional properties, such as reactive oxygen species production, antioxidant response, calcium homeostasis, and mitochondrial membrane potential. Our results demonstrate that hyperglycaemic-like condition *per se* is well-tolerated by rMC-1 cells but makes them more susceptible to a pro-inflammatory environment, exacerbating the effects of this stressful condition. More specifically, rMC-1 cells exposed to high glucose decrease their ability to counteract oxidative stress, with consequent toxic effects. In conclusion, our study offers new insights into Müller cell pathophysiology in DR and proposes a novel *in vitro* model which may prove useful to further investigate potential antioxidant and anti-inflammatory molecules for the prevention and/or treatment of DR.

## Introduction

Diabetic retinopathy (DR) is the most common microvascular complication of diabetes and represents a leading cause of vision loss. The risk to develop DR during a lifetime is estimated to be over 90% in patients affected by type 1 diabetes (T1D) and about 50%–60% in patients with type 2 diabetes (T2D). Although DR is traditionally considered a microvascular disease, growing evidence indicates that neurodegeneration can also occur early in disease progression, even though without a clear relationship and a defined temporal correlation between vascular and neuronal dysfunction (Lieth et al., 2000; Wong et al., 2016; Antonetti et al., 2021).

The disease develops slowly and is characterized by two overlapping clinical stages: non-proliferative DR (NPDR), going from mild to moderate, up to severe; and proliferative DR (PDR), characterized by neovascularization. Histopathological hallmarks of NPDR include thickening of capillary basement membranes, loss of pericytes, and formation of microaneurysms, which are often surrounded by small ischemic areas. As ischemia progresses, an abnormal growth of new retinal blood vessels occurs; this PDR stage is often associated with an increased permeability of retinal capillaries, which causes the formation of hard exudates and edema. If edema is concentrated around the macula it is called diabetic macular edema (DME); it can be detected in all stages of DR and contributes to visual loss, together with retinal detachment and hemorrhages (Wong et al., 2016; Forrester et al., 2020; Antonetti et al., 2021). Vascular endothelial growth factor (VEGF) is overexpressed by several retinal cell types (mainly pericytes, Müller cells, astrocytes, and endothelial cells) in response to hypoxic conditions and appears to play a central role in microvascular retinal alterations, including an increase in vascular permeability and endothelial cell proliferation, therefore promoting macular edema (Stewart et al., 2000; Mrugacz et al., 2021).

Besides ischemia, also inflammatory processes are associated with microvascular complications in DR. Inflammatory mediators are released by vessels, due to early vascular dysfunction, and by glial cells (mainly Müller cells and microglia), in response to neurodegeneration and environmental alterations. Moreover, chronic retinal inflammation results in leukocyte activation and adhesion to vascular endothelium, thereby contributing to vessel occlusion and blood-retinal barrier (BRB) damage (Nguyen et al., 2009; Wong et al., 2016; Forrester et al., 2020). Numerous inflammatory factors have been found in vitreous fluids and several chemokines (CXCL16 and CX3CL1), cytokines (IL-1β, IL-6, IL-8, IL-10, TNF-α), growth factors (VEGF and TGF-β), as well as adhesion molecules (VCAM-1 and ICAM-1) and monocyte chemo attractant protein-1 (MCP-1) are reported to contribute to retinopathy pathogenesis (Al-Kharashi, 2018; Abu El-Asrar et al., 2021; Antonetti et al., 2021; Mrugacz et al., 2021).

A central role in the development of DR is also played by oxidative stress: high glucose causes an increase in reactive oxidative species (ROS), by direct sugar metabolism and by the formation of advanced glycation end products (AGEs), that, in turn, promote a further increase in ROS levels. Moreover, a high amount of polyunsaturated fatty acids makes the retina highly susceptible to the oxidative environment (Kowluru and Chan, 2007; Santos et al., 2011). Finally, the role of oxidative stress in DR is supported by the evidence that antioxidant molecules can prevent mitochondria dysfunction and apoptosis of retinal cells (Kowluru and Odenbach, 2004).

All these retinal alterations affect the function of the neurovascular unit, formed by ganglion cells, glia and blood vessels, contributing to the neurodegenerative process and DR progression (Wong et al., 2016; Forrester et al., 2020).

Müller cells represent the most abundant retinal macroglial cells; they span radially the entire thickness of the retina, constitute the support of the retinal structure, and are the core of the neurovascular unit (Antonetti et al., 2006; Nian et al., 2021). Due to their unique morphology and localization, interposed between retinal neurons and blood vessels, Müller cells not only provide nutrients and metabolic support to surrounding neurons but also redistribute and buffer metabolic waste, potassium, and neurotransmitters, along their long processes (Vecino et al., 2016; Subirada et al., 2018). Their essential role in the healthy neuroretina can be altered in DR; indeed, it is well known that the diabetic condition promotes Müller cell activation, a process characterized by the expression of GFAP and gliosis (Bringmann et al., 2006). *In vitro* studies demonstrated that, upon activation, Müller cells release growth factors, chemokines, and cytokines (Eastlake et al., 2016; Rezzola et al., 2021; Schmalen et al., 2021), most of which are reported to play detrimental effects toward retinal cells implied in DR pathology (Antonetti et al., 2006; Mesquida et al., 2019). Therefore, Müller cells are expected to contribute significantly to the alteration of BRB integrity and to the processes of neovascularization, retinal gliosis, and chronic inflammation.

In this study, we characterize the functional changes occurring in a rat Müller cell line (rMC-1), in the presence of high extracellular glucose concentration combined with inflammatory and oxidative conditions, to recapitulate the pathological environment typical of DR.

Our results indicate that high levels of extracellular glucose are well tolerated by Müller cells; however, this condition exacerbates the toxic effects of pro-inflammatory and oxidative insults, in terms of the activation process, calcium dysregulation, and oxidative stress production.

## Materials and Methods

### Materials

Cell culture media and reagents, if not otherwise stated, were from Gibco (Thermo Fisher Scientific, Carlsbad, CA, USA). Plates and flasks were from Nalgene Nunc (Rochester, NY, USA). Chemicals, if not otherwise specified, were from Tocris (Bristol, UK) or Merck-Sigma (Darmstadt, Germany).

### Cell Culture

Immortalized rat retinal Müller cells (rMC-1; Aurogene) were cultured in Dulbecco’s Modified Eagle Medium (DMEM, Sigma-Aldrich) containing either 5 mM glucose (NG, normal glucose) or 35 mM glucose (HG, high glucose), supplemented with 10% fetal clone III serum (FCIII; Hyclone, South Logan, UT, USA), 100 U/ml of penicillin, 100 mg/ml streptomycin (Sigma-Aldrich). Cultures were maintained at 37°C in a 5% CO_2_ humidified incubator.

### Cell Treatments

To induce rMC-1 activation, cells were treated overnight with a mix, directly added to the culture medium, of the following proinflammatory cytokines (CKs): IL-1β (10 ng/ml), TNF-α (30 ng/ml) and IFN-γ (20 ng/ml). Where indicated, rMC-1 cells were subjected to an acute treatment with hydrogen peroxide (H_2_O_2_) 300 μM, for 15 min, freshly prepared in Krebs-Ringer-Hepes (KRH) buffer. ATP was used at 100 μM dissolved in KRH.

### Measurement of Cell Proliferation

Cell proliferation was measured through the 3-(4,5-dimethylthiazol-2-yl)-2,5-diphenyltetrazolium bromide (MTT) assay. Cells were grown in 96-well plates for 24/48 h and then incubated with MTT (0.5 mg/ml; Sigma Aldrich), freshly prepared in KRH, for 1 h. Supernatants were removed, the formazan precipitates were dissolved in dimethyl sulfoxide (DMSO) and the absorbance was then read at 570 nm by a microplate reader (Bio-Rad, Hercules, CA, USA).

### Measurement of Nitric Oxide Release

Nitric oxide (NO) production was determined by measuring the accumulation of nitrite in the culture medium. Nitrite was assayed colorimetrically by a diazotization reaction using the Griess reagent, composed of a 1:1 mixture of 1% sulfanilamide in 5% orthophosphoric acid and 0.1% naphtylenethylenediamine dihydrochloride in H_2_O. Cells were grown in 12-well plates and treated overnight with a mix of CKs; culture medium (100 μl) was collected and mixed with Griess reagent (100 μl) in a 96-multiwell plate and the optical density at 570 nm was measured within 10 min. The nitrite concentration in the samples was interpolated from a NaNO_2_ standard curve, ranging from 0 to 100 μM and normalized for the protein content.

### Western Blotting

Cells were washed with phosphate-buffered saline and lysed for 10 min at 4°C with lysis buffer (2% NP40, 0.2% SDS, 0.01 M EDTA/Na, protease inhibitor cocktail). Samples (30 μg of proteins per lane) were then separated by standard SDS–polyacrylamide gel electrophoresis (SDS-PAGE) and electrically transferred onto nitrocellulose membrane. After 1 h of blocking with TBST (10 mM Tris/HCl, 150 mM NaCl, 0.1% Tween-20) containing 5% skimmed powdered milk, the membranes were incubated overnight with the primary antibodies and, after extensive washing, with horseradish peroxidase-conjugated anti-mouse secondary antibody (Bio-Rad, Hercules, CA, USA). For loading controls. membranes were stripped in acidic buffer (0.2 M glycine, 0.1% SDS, 1% Tween-20, pH 2.2) and re-probed with the appropriate antibody. Proteins were revealed by direct acquisition using the Bio-Rad Chemidoc system by Super Signal West Chemiluminescent Substrate (ThermoFisher Scientific, Waltham, MA, USA). Bands were quantified using Image Lab software (Bio-Rad, Hercules, CA, USA) and protein levels normalized against the loading control. The following primary antibodies were used for the experiments: Mouse anti-alpha-tubulin 1:6,000 (T9026, Merck-Sigma) and mouse anti-iNOS 1:1,000 (610328, iNOS/NOS Type II, Becton Dickinson).

### Cytokine/Chemokine Profile Analysis

The cytokine/chemokine profile analysis was performed according to manufacturer instructions (RayBio^®^ C-Series Rat Cytokine Antibody Array C1, RayBiotech Life, Inc., Georgia, USA); to assess a possible cross-reaction with the human or mouse cytokines used to promote the activation process, a sample of medium spiked with the CK mix was analyzed and the obtained value was subtracted from experimental data.

### Single-Cell Analyses

#### Solutions and Dye Loading

Single cells experiments were performed in Krebs-Ringer-Hepes buffer (KRH - 5 mM KCl, 125 mM NaCl, 2 mM CaCl_2_, 1.2 mM MgSO_4_, 1.2 mM KH_2_PO_4_, 6 mM glucose and 20 mM Hepes, pH 7.4). Fluorescent dyes (from Molecular Probes, Invitrogen, Carlsbad, CA, USA, when not specified) were dissolved and loaded as follows: (a) fura-2 acetoxymethyl ester (Calbiochem): 40 min at 37°C, 4 μM final concentration; (b) 5-(and-6)-chloromethyl-2’,7’-dichlorodihydrofluorescein diacetate, acetyl ester (CM-H_2_DCFDA): 30 min at 37°C, 5 μM final concentration; (c) CellROX Orange Reagent (5 μM, 30 min; Invitrogen, Thermo Fisher Scientific); (d) Hoechst 33342: 5 min at room temperature, 2 μg/ml final concentration; (e) Tetramethyl rhodamine methyl ester (TMRM): 15 min at room temperature (and maintained in the bath during the experiment), 25 nM final concentration; (f) fluo-8 (40 min at 37°C, 4 μM final concentration). After dye loading, cells were washed twice with fresh KRH and kept in the same buffer.

#### Videomicroscopy Setup

A video imaging setup consisting of an Axioskope 2 microscope (Zeiss, Oberkochen, Germany) and a Polychrome IV (Till Photonics, GmbH, Martinsried, Germany) light source was used for single-cell experiments. Fluorescence images were collected by a cooled CCD videocamera (PCO Computer Optics GmbH, Kelheim, Germany). The “Vision” software (Till Photonics) was used to control the acquisition protocol and to perform data analysis (Vangelista et al., 2005; Codazzi et al., 2006). This instrument was used for both fura-2-based calcium analyses and mBCl-based GSH measurements.

The automated ArrayScan XTI platform (Thermo Fisher Scientific) was used for fluo-8-based calcium measurements, reactive oxygen species (ROS), and TMRM-mitochondrial membrane potential analyses.

#### Design of Experiments (DoE)

Design of Experiments (DoE) is a mathematical method used to plan, analyze and interpret tests with multivariable parameters. DoE allows to identify the factors and their interactions that influence the experimental output (Mancinelli et al., 2021); in this study, the DoE approach has been used to optimize the experimental design of single-cell analyses described below and to select the most suitable parameters to be included in the experimentation.

#### ROS and Mitochondrial Membrane Potential Measurements

rMC-1 cells, at rest or upon CK-activation, were plated on 96-well plate the day before videomicroscopy image acquisition. Fluorescent dyes CM-H_2_DCFDA and CellROX Orange were used to analyze ROS accumulation in the cytosol and mitochondria, respectively. TMRM was used to analyze mitochondrial membrane potential while nuclear staining, necessary for image acquisition and analysis, was performed with Hoechst 33342. Eleven fields/well were acquired.

#### Calcium Measurement

Two different Ca^2+^ measurements were performed. The first method consisted of the incubation of cells with ratiometric dye fura-2: excitation wavelengths of 340 and 380 nm were used and 340:380 fura-2 ratio was calculated as mean values within ROIs drawn in cells. The second one was based on the loading of cells with fluo-8 analyzed by the ArrayScan XTI platform; the automatic system acquires a single image per well (at its middle point). After the acquisition of four images of basal fluorescence, the stimuli, previously added in another plate, are automatically injected in the cells. Particularly, the system injects 50 μl of stimulus at a speed of 50 μl/s. For the analyses, only responsive cells were considered (i.e., the cells showing a fold increase higher than 0.5 value).

#### Glutathione Measurement

Reduced Glutathione (GSH) value was measured at the single-cell level, by loading the rMC-1 cells with 50 μM monochlorobimane (mBCl). The kinetics of fluorescent mBCl conjugate formation was followed for 30 min.

### Data Analysis

Data are presented as mean ± SEM as specified. Statistical significance was tested using two-way ANOVA followed by Tukey *post-hoc* tests, Mann-Whitney test, or multiple unpaired t-test for values normally distributed. Statistical analysis was performed using GraphPad Prism v.9 (GraphPad Software, San Diego, CA, USA).

## Results

### Effect of High Glucose on Müller Cell Line (rMC-1) Proliferation and Activation

To explore the effects of elevated glucose in DR-like retinal microenvironment, we used an immortalized cell line obtained from rat adult retina (rMC-1), which is known to recapitulate the phenotype of primary Müller cells (Sarthy et al., 1998).

rMC-1 cells were maintained in culture under physiological extracellular glucose concentration (5 mM; Normal Glucose—NG) or exposed to a high glucose-containing medium (35 mM; High Glucose—HG) for 24–48 h.

**Figure 1:**
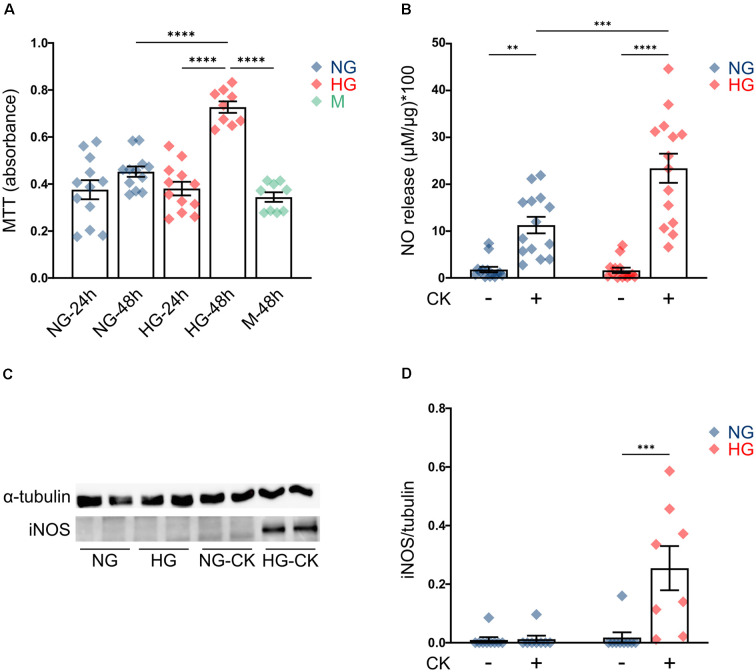
Effect of high glucose (HG) on rMC-1 cellular proliferation and cytokine-induced activation. **(A)** Scatter graph with bars represents the proliferation rate of rMC-1 cells, plated at a density of 60,000 cells/well and examined after 24 or 48 h. HG promoted rMC-1 cellular proliferation at 48 h, but not at 24 h. Absorbance values of MTT [3-(4,5-dimethylthiazol-2-yl)-2,5-diphenyltetrazolium bromide] are expressed as mean ± SEM of four independent experiments. Statistical significance is calculated by two-way ANOVA followed by Tukey’s *post-hoc* test (****p < 0.0001). **(B)** Scatter graph with bars represents nitric oxide (NO) release from rMC-1 cells, indicative of cellular activation. rMC-1 cells were plated and maintained in culture for 24 h, then treated for 24 h with a cytokine mix (IL-1β, TNF-α, and IFN-γ). 48 h after plating, rMC-1 cells were lysed, and NO concentration was normalized over their total protein content. Absorbance values are expressed as mean ± SEM of five independent experiments. Statistical significance is calculated by two-way ANOVA followed by Tukey’s *post-hoc* test (**p < 0.005; ***p < 0.001; ****p < 0.0001). **(C)** Representative western blot image of inducible NO synthase (iNOS) expression of rMC-1 cells. iNOS is detectable only in HG-maintained rMC-1 cells treated with the cytokine mix. **(D)** Scatter graph with bars representing iNOS expression of untreated and CK-treated rMC-1 cells normalized over α-tubulin expression. Fold values are expressed as mean ± SEM of five independent experiments. Statistical significance is calculated by two-way ANOVA followed by Tukey’s *post-hoc* test (***p < 0.001). NG, normal glucose; HG, high glucose; M, mannitol; CK, cytokines.

First, we evaluated the effect of HG on rMC-1 cell proliferation, by measuring cellular metabolic activity (MTT assay); cells maintained a constant proliferation rate in NG (at both 24 and 48 h), while, after prolonged exposure to HG (48 h), showed a significant increase in cell growth, compared to both cells exposed to the shorter treatment (HG, 24 h) and cells grown in NG (Figure 1A). Treatment with the same concentration of mannitol (35 mM), a metabolically inert sugar, had no effect on the proliferation rate (Figure 1A).

This result is in line with the behavior of Müller cells *in vivo*, under diabetic conditions, where chronic exposure to HG activates proliferative pathways and promotes gliosis (Coughlin et al., 2017; Li et al., 2020). Since studies performed in human tissue and animal models showed that HG can promote activation of Müller cells (Mizutani et al., 1998; Gerhardinger et al., 2005), we investigated whether HG was able to induce, *per se*, the activation of rMC-1 cells or to amplify the effect of pro-inflammatory conditions. rMC-1 cells were maintained for 48 h in NG or HG and treated, for the last 24 h, with a mix of cytokines (rat IL-1β (10 ng/ml), human IFN-γ (20 ng/ml), and human TNF-α (30 ng/ml). This CK mix was chosen based on both data in the literature (Schmalen et al., 2021) and our previous experience with glial cell activation (Macco et al., 2013; Pelizzoni et al., 2013). Nitric oxide (NO) release and inducible nitric oxide synthase (iNOS) expression were assessed as glial cell activation markers (Lazzaro et al., 2014). While HG treatment alone was not sufficient to induce NO release (Figure 1B, HG untreated -UNT), CK administration promoted a release of NO, which was further reinforced when HG treatment was combined (Figure 1B). As expected, NO release was accompanied by the expression of iNOS, which was detectable only in rMC-1 cells maintained in HG and treated with the mix of CKs (Figures 1C,D). These results suggest that HG can exacerbate the activation response of the Müller cell line.

### Effect of CK Treatment on HG-Induced Chemokine and Cytokine Release

**Figure 2:**
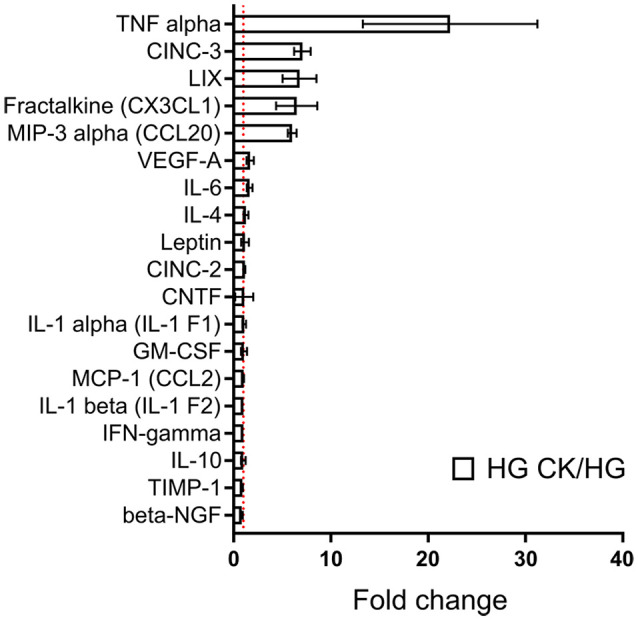
Effect of HG on cytokine and chemokine release profile in CK-activated rMC-1 cells. Bar plot represents cytokines and chemokines profile of rMC-1 cells exposed to HG and eventually treated with the CK mix (HG CK); the results are shown as values obtained from HG CK condition normalized over HG. Fold values are expressed as mean ± SEM of three independent experiments.

Considering the synergistic effect induced by the concomitant exposure to HG and CK on NO release, we investigated whether this condition could also influence the capability of rMC-1 cells to release cytokines and chemokines, a process that highly contributes to the inflammatory condition in DR. rMC-1 cells were maintained for 48 h in HG and eventually treated (during the last 24 h), with CKs. In these experiments, human IL-1β was employed to discriminate between the cytokines used as pro-inflammatory stimuli and those released by rMC-1 cells and measured with a semi-quantitative rat-specific array. Figure 2 shows that the HG-CK treatment, compared with HG alone, strongly increased the secretion of TNF-α (more than 20-fold) as well as of several chemokines [in particular Cytokine-Induced Neutrophil Chemoattractant-3 (CINC-3), fractalkine (CX3CL1), Lipopolysaccharide-induced CXC chemokine (LIX) and Macrophage Inflammatory Protein-3α (MIP-3α/CCL20)], therefore indicating a contribution of Müller cells to pro-inflammatory conditions.

### Calcium Homeostasis in rMC-1 Cells Under HG, Inflammatory, and Oxidative Conditions

**Figure 3:**
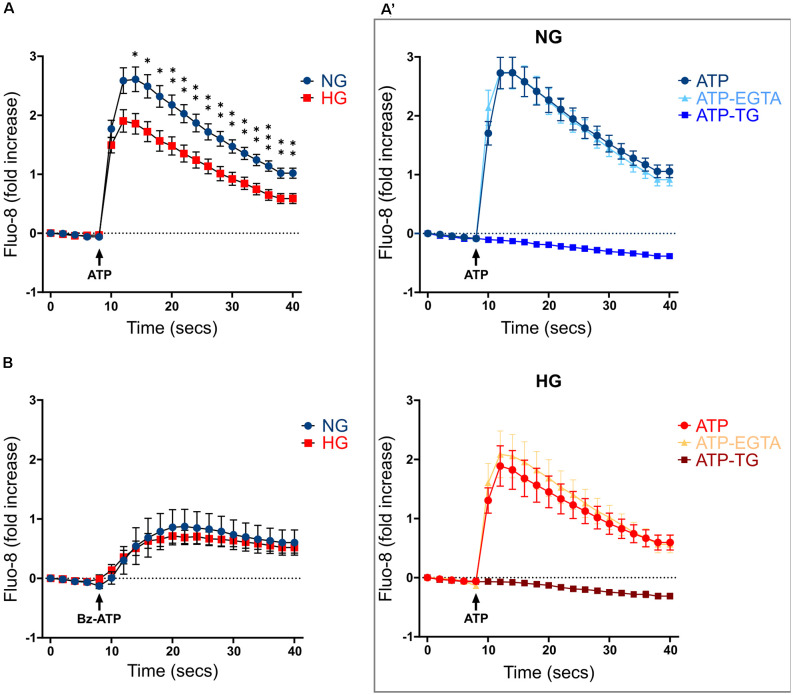
Calcium responses to ATP stimulation in NG and HG. **(A)** Kinetics of Ca^2+^ responses in responding rMC-1 cells following ATP acute stimulation. ATP (100 μM) was added through an automated liquid handling system to rMC-1 cells after acquiring basal Ca^2+^ level images. Values are represented as fold increase with respect to their basal Ca^2+^ level. Fold increase values are expressed as mean ± SEM of three independent experiments. Statistical significance is calculated by unpaired two-tailed Student’s t-test (*p < 0.05; **p < 0.005; ***p < 0.001). **(A’)** Kinetics of Ca^2+^ responses in both NG- and HG-maintained rMC-1 cells stimulated with ATP alone or in the presence of either EGTA (3 mM) or thapsigargin (TG, 1 μM). Stimuli were added to rMC-1 cells through an automated liquid handling system after acquiring basal Ca^2+^ level images. Values are represented as a fold increase over their basal Ca^2+^ level. Fold increase values are expressed as mean ± SEM of three independent experiments. **(B)** Kinetics of Ca^2+^ responses in rMC-1 cells stimulated with Bz-ATP. Bz-ATP (300 μM) was added through an automated liquid handling system to rMC-1 cells after acquiring basal Ca^2+^ level images. Values are represented as a fold increase over their basal Ca^2+^ level. Fold increase values are expressed as mean ± SEM of three independent experiments.

**Figure 4:**
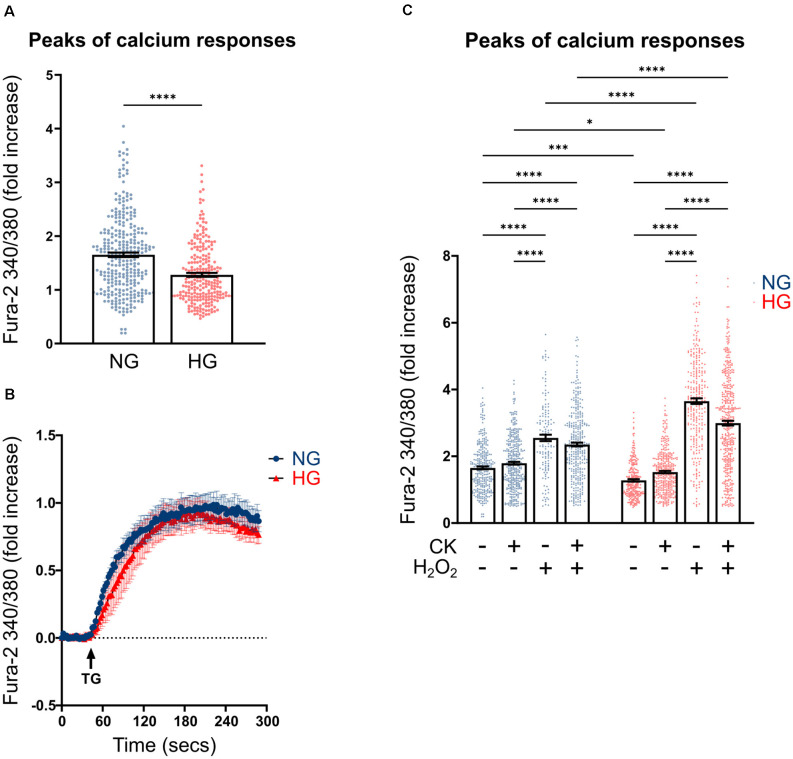
Effect of CK treatment and oxidative stress on ATP-mediated calcium responses. **(A)** Scatter graph with bars represents the peaks of calcium response following ATP acute stimulation. Each dot represents the maximum peak of a single rMC-1 cell, from five separate experiments. ATP (100 μM) was manually added to rMC-1 cells after acquiring basal Ca^2+^ level images. Values are represented as a fold increase over their basal Ca^2+^ level. Fold increase values are expressed as mean ± SEM. Statistical significance is calculated by the non-parametric Mann–Whitney U test (****p < 0.0001). **(B)** Kinetics of Ca^2+^ response in rMC-1 cells following thapsigargin (1 μM, TG) acute stimulation. Values are represented as a fold increase over their basal Ca^2+^ level. Fold increase values are expressed as mean ± SEM of three independent experiments. **(C)** Scatter graph with bars represents the different peaks of calcium responses following ATP (100 μM) acute stimulation. Each dot represents the maximum peak of a single rMC-1 cell. H_2_O_2_ (300 μM) was added 15 min before the experimental acquisition. Values are represented as a fold increase over their basal Ca^2+^ level. Fold increase values are expressed as mean ± SEM of 3–5 independent experiments. Statistical significance is calculated by two-way ANOVA followed by Tukey’s *post-hoc* test (*p < 0.05; ***p < 0.001; ****p < 0.0001).

Previous studies carried out on both primary microglia from diabetic mice (Portillo et al., 2017) and immortalized lines of endothelial cells and pericytes (Platania et al., 2017, 2019), proposed the HG-induced activation of the purinergic ionotropic receptor P2X_7_ (P2X_7_R) as a key step for the release of IL-1β, one of the main cytokines involved in DR. Given this premise, we investigated the calcium response to ATP in rMC-1 cells and its potential modulation by HG and inflammatory conditions. rMC-1 cells were maintained for 48 h in NG- or HG-containing medium, then loaded with the calcium-sensitive dye fluo-8 and analyzed by a high throughput microscopy setup. Surprisingly, calcium response to ATP (100 μM) was significantly lower in rMC-1 cells maintained in HG than in NG (Figure 3A). In both conditions, the increase in intracellular calcium, upon ATP administration, was unaffected by the presence of an extracellular calcium chelator (EGTA, 3 mM), while it was completely prevented by the depletion of intracellular calcium stores with thapsigargin (1 μM), a blocker of sarco/endoplasmic reticulum Ca^2+^-ATPases (SERCA; Figure 3A’). Overall, these results pointed to the major involvement of ATP-activated metabotropic, rather than ionotropic, receptors. This deduction received confirmation when only a moderate increase in intracellular calcium was observed upon stimulation with BzATP, a selective P2X_7_R agonist, even at its maximal concentration (300 μM; Figure 3B). This analysis was refined at the single-cell level, by fura-2 ratiometric measurement of the intracellular calcium concentration. Peak values of calcium responses to 100 μM ATP, shown in Figure 4A, confirmed that exposure to HG-induced a significant reduction in the amplitude of calcium elevation when compared to NG. This effect was not the consequence of lower loading of the intracellular calcium stores, since acute administration of thapsigargin (1 μM) promoted comparable calcium release (Figure 4B), this result also suggests that rMC-1 cells have an elevated capability to control calcium homeostasis, even in presence of high glucose concentration.

We next investigated whether inflammatory conditions affected calcium responses; intracellular calcium levels were analyzed in rMC-1 cells, maintained for 48 h in NG or HG, and subjected to the activation protocol during the last 24 h. CK treatment slightly increased the ATP-mediated calcium responses in HG conditions, while it did not show any effect on NG-maintained cells (Figure 4C). Another neurotoxic condition typically observed in DR is the presence of high oxidative stress levels; to mimic this condition, activated rMC-1 cells were acutely exposed (15 min before starting calcium measurement) to hydrogen peroxide (H_2_O_2_, 300 μM). Oxidative stress significantly raised the amplitude of the ATP-mediated calcium response in NG- and even more (almost four times) in HG-exposed cells. The massive calcium responses to ATP observed in HG and oxidative conditions prevented any additional effect of CK treatment (Figure 4C).

These data suggest that calcium homeostasis in the Müller cell line is well controlled in HG, but it is strongly altered, compared to NG, by pro-inflammatory conditions and even more by an oxidative environment.

### Oxidative Stress Production in rMC-1 Cells

**Figure 5:**
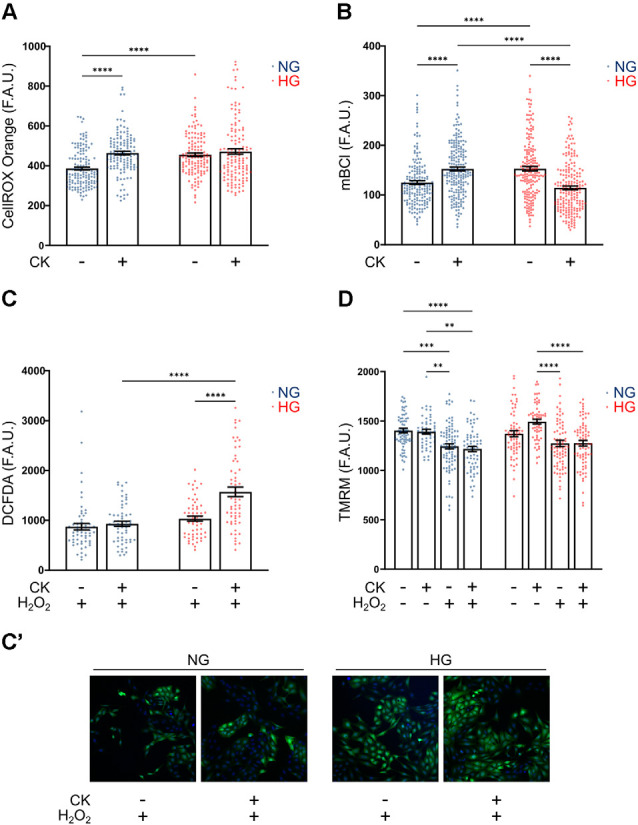
Effect of CK treatment and oxidative stress on cellular redox state and mitochondrial membrane potential. **(A)** Scatter graph with bars represents CellROX Orange fluorescence values relative to ROS production in resting and CK-treated rMC-1 cells. Values are expressed as mean ± SEM of three independent experiments. Statistical significance is calculated by two-way ANOVA followed by Tukey’s *post-hoc* test (****p < 0.0001). **(B)** Scatter graph with bars represents mBCl fluorescence values relative to reduced glutathione (GSH) content in resting and CK-treated rMC-1 cells. Values are expressed as mean ± SEM of three independent experiments. Statistical significance is calculated by two-way ANOVA followed by Tukey’s *post-hoc* test (****p < 0.0001). **(C)** Scatter graph with bars represents DCFDA fluorescence values relative to ROS production in resting and CK-treated rMC-1 cells with a pro-oxidative stimulus (H_2_O_2_, 300 μM). Values are expressed as mean ± SEM of three independent experiments. Statistical significance is calculated by two-way ANOVA followed by Tukey’s *post-hoc* test (****p < 0.0001). **(C’)** Representative images of **(C)**. **(D)** Scatter graph with bars represents TMRM fluorescence values relative to mitochondrial membrane potential in untreated and CK-treated rMC-1 cells with or without a pro-oxidative stimulus (H_2_O_2_, 300 μM). Values are expressed as mean ± SEM of three independent experiments. Statistical significance is calculated by two-way ANOVA followed by Tukey’s *post-hoc* test (**p < 0.005; ***p < 0.001; ****p < 0.0001).

Based on the previous results, we investigated whether rMC-1 cells could directly contribute to oxidative stress by producing reactive oxygen species (ROS), under either basal or activated conditions. In these experiments, we used CellROX Orange, a mitochondrial probe whose fluorescence depends on ROS-mediated oxidation. While NG CK treatment caused an increase in ROS production, the HG condition was sufficient *per se* to promote a comparable ROS elevation that was not further affected by CKs (Figure 5A).

Since the main antioxidant defense in glial cells is represented by reduced glutathione (GSH), we evaluated the levels of GSH under the conditions described above. rMC-1 cells were loaded with monochlorobimane (mBCl), a probe that becomes fluorescent upon conjugation with GSH, and the fluorescence was quantified after 30 min, when the reaction reached the plateau phase. rMC-1 cells exposed to HG showed a higher GSH level compared to normal conditions, as a possible compensatory mechanism; however, upon activation, GSH was consumed by HG-cells, most likely to counteract ROS increase under inflammatory conditions (Figure 5B). We, therefore, investigated whether the reduced antioxidant defenses of HG-activated cells could prevent further protection in presence of additional oxidative insults. For this purpose, we utilized DCFDA, another ROS fluorescent probe that is sensitive also to hydrogen peroxide (H_2_O_2_). When rMC-1 cells were acutely treated with H_2_O_2_ (15 min, 300 μM), the HG-activated cells showed a marked rise of ROS accumulation, when compared to both non-activated and NG counterparts (Figures 5C,C’). These results indicate again that, although rMC-1 cells are resistant to HG, this condition worsens their susceptibility to an inflammatory and oxidative environment.

Mitochondria represent the main site of ROS production; indeed, alterations of these organelles can contribute to ROS accumulation, while oxidative stress, in turn, amplifies mitochondrial damage. Staining with TMRM, a fluorescent dye that accumulates in healthy mitochondria with preserved membrane potential, did not reveal marked morphological alterations (not shown) or membrane potential reduction under HG and inflammatory conditions (i.e., in the presence of CKs; Figure 5D). On the other hand, treatment with H_2_O_2_ (15 min, 300 μM), caused in both NG and HG a significant reduction of TMRM fluorescence that was not further affected by CK treatment. This reduction in mitochondrial membrane potential, comparable at both glucose concentrations, further highlights the susceptibility of Müller cells to oxidative insults.

## Discussion

Diabetic retinopathy (DR) has long been regarded as a microvascular complication of diabetes mellitus, but an increasing body of evidence points the attention also to a neurodegenerative component, mainly affecting the ganglion cells and detectable from the early stages of the disease. Within this complex framework, little attention has been devoted to Müller cells, which make contact with virtually every cell type composing the neuroretina. It follows a “Janus effect”: Müller cells can efficiently support surrounding neurons, but their functional alteration can seriously impact neuronal survival.

In this study, we investigated potential functional alterations of Müller cells under experimental conditions that mimic those observed in DR patients (i.e., hyperglycemia, oxidative stress, and inflammation). The use of the rMC-1 cell line allowed us to recapitulate the properties of the rat retinal Müller cells (Sarthy et al., 1998), thus avoiding the complications caused by the low efficiency in the recovery of primary cells from the retina as well as their tendency to transdifferentiate into myofibroblast-like cells after 1–2 weeks *in vitro*, with ensuing profound changes in their proteomic profile (Guidry, 1996).

Our results, which indicate an ameliorated viability of rMC-1 cell line exposed to HG, are in agreement with data published by some authors (Lu et al., 1999; Vellanki et al., 2016) but in contrast with those of others, who report increased cell death (Du et al., 2004; Muto et al., 2014; Ma et al., 2020). The discrepancy between the results may be accounted for slight differences in cell culture conditions, and in the protocols of glucose concentration change. A similar situation is also reported on primary Müller cell cultures, with evidence supporting either a proliferative (Sun et al., 2013) or a toxic effect of HG concentrations (Zhao et al., 2015).

Upregulation of GFAP, a distinctive marker of gliosis, is the typical response of Müller cells to virtually any pathologic insult and is reported to occur early during DR pathogenesis (Barber et al., 2000; Bogdanov et al., 2014). However, *in vitro* data obtained in the rMC-1 cell line appear more contradictory. Our results show a lack of GFAP immunoreactivity in all conditions analyzed, while other studies describe a marked GFAP expression under HG conditions (Picconi et al., 2019; Ma et al., 2020). Also, primary Müller cells are reported to increase GFAP expression in HG conditions (Matteucci et al., 2014), even though the immunoreactivity appears to be heterogenous, a condition that might reflect the phenotypic diversity of Müller cells in culture (Vecino et al., 2016). This raises the question of whether the *in vitro* GFAP expression is representative of the *in vivo* reactive state in response to inflammatory and toxic insults, or other typical markers of glial activation are to be considered to better describe this condition (Consonni et al., 2011; Macco et al., 2013). Induction of iNOS expression, a typical marker of glia activation, was observed after exposure of rMC-1 cells to some of the DR-characterizing cytokines (IL-1β, TNF-α, IFN-γ; Boss et al., 2017), but not to HG. However, HG was able to exacerbate the activation process promoted by pro-inflammatory CKs. It should be recalled that iNOS induction leads to an increase in NO release that favors the reaction with superoxide to form peroxynitrite, a powerful oxidant that causes oxidation of membrane phospholipids, inactivation of sulfhydryl-containing enzymes, DNA fragmentation, and impairment of mitochondrial energy production (Forrester et al., 2020).

The competence of HG to exacerbate the retinal inflammatory response, by potentiating the release of chemokines from CK-activated rMC-1 cells, deserves further attention. In fact, the release of chemokines involved in the recruitment of macrophages—such as TNF-α (highly detected in NPDR patients; Boss et al., 2017), CINC-3, CXC3CL1, LIX, and MIP-3α is potentiated, while the levels of neuroprotective factors—such as ciliary neurotrophic factor (CNTF), beta nerve growth factor (β-NGF) or tissue inhibitor of metalloproteinase 1 (TIMP-1)—are unchanged. Interestingly, also VEGF release is not affected by HG upon CK-treatment, suggesting that Müller cells are not involved in the detrimental neo-angiogenic process. Unexpectedly, also IL-1β secretion was not affected by HG and pro-inflammatory cytokines, prompting a careful reconsideration of the contribution of reactive Müller cells in the production of one of the main pro-inflammatory cytokines detected in DR patients (Boss et al., 2017). Indeed, it is recognized that several cell types, including pericytes, endothelial and microglial cells (Platania et al., 2017, 2019; Portillo et al., 2017), can release IL-1β following the stimulation of NLRP3 inflammasome activity, with ensuing processing of pro-IL-1β to its mature form. An important player in this pathway is the ionotropic purinergic P2X_7_ receptor that, by responding with a calcium elevation to the high levels of extracellular ATP present in the pathological environment of the DR retina, triggers NLRP3 inflammasome activation (Andrejew et al., 2020; Paik et al., 2021). However, this pathway does not seem pivotal in Müller cells, given the little extent of intracellular calcium responses induced by selective stimulation of the P2X_7_ receptor in rMC-1 cells (Yu et al., 2009). We provide evidence along the same line, showing that ATP, the physiological and broad agonist of the P2 receptor family, elicits calcium responses that solely depend on intracellular stores, thus pointing to a role for metabotropic, rather than ionotropic, purinergic receptors. Overall, the lack of effect of HG on both IL-1β release and P2X_7_-mediated calcium responses should lead to reconsidering the impact of this pathway in Müller cells as well as the relevance of therapeutic strategies based on antagonizing P2X_7_ receptors on these cells.

Some further observations are worth making here on the calcium responses to acute ATP stimulation. First, the peaks of calcium elevation were surprisingly higher in NG than in HG. This lack of responsivity in HG cannot be ascribed to a different Ca^2+^ storage capacity, rather, it might reflect the positive effect of HG on the activity of PKC described in DR (Shiba et al., 1993; Das Evcimen and King, 2007); indeed, the activated PKC might contribute to downregulate Ca^2+^ signaling by a phosphorylation-mediated inactivation of metabotropic receptors (Kawabata et al., 1998; Codazzi et al., 2001) and phospholipase C (Yue et al., 2000).

Second, calcium responses to ATP were potentiated in the presence of inflammatory conditions and, even more, of an oxidative environment, in agreement with the knowledge that an elevation of intracellular ROS can severely affect calcium handling (Pelizzoni et al., 2008; Feno et al., 2019). In HG, the effect of hydrogen peroxide on ATP-mediated calcium elevation was significantly more pronounced than in NG, in line with a different capability of rMC-1 cells to control their oxidative stress under normal or DR mimicking conditions. In fact, in line with other published data (Ma et al., 2020; Kida et al., 2021), HG-rMC-1 cells showed a higher basal level of ROS compared to NG-rMC-1 cells; this promotes a compensatory increase in GSH level that, however, appears to be consumed by the cells to contain further ROS elevation upon CK treatment (that instead occurs in NG-cells; Bettegazzi et al., 2019). Consequently, HG-rMC-1 cells become far less competent than NG-rMC-1 cells to counteract an elevation of oxidative stress in the extracellular environment, with an ensuing marked increase in intracellular ROS elevation (Codazzi et al., 2016). On the other hand, a physiological mitochondrial membrane potential (Δψm) is a sign of a healthy cell, since Δψm not only provides the charge gradient necessary for ATP production (De Stefani et al., 2016) but also regulates ROS production. A decrease in mitochondrial membrane potential indicates a bioenergetic stress and may result in the release of apoptotic factors leading to cell death. A partial loss of Δψm was observed only in the presence of oxidative condition, both in NG and HG, while cell activation did not appear to be influential.

Oxidative conditions, a consequence of the metabolic abnormalities caused by hyperglycemia, seem to exert the most prominent effect on functional parameters of Müller cells, causing not only ROS increase and mitochondrial alterations but also a marked dysregulation in calcium responses. The potential downstream effects are numerous, ranging from modifications of physiological interactions established with ganglion neurons and virtually all cell types in the retinal tissue, to a threat to Müller cell survival itself, laying the foundations for future *in vivo* studies.

Altogether, although limited to an *in vitro* model of Müller cells, our experiments provide new insights into the relationship between Müller cells and the typical DR environment. While the attention is principally focused on HG as the main toxic mediator in DR pathogenesis, HG condition seems to be well tolerated by the rMC-1 Müller cell line. However, HG appears to exacerbate pre-existing pro-inflammatory and oxidative conditions, acting as a toxicity enhancer. Finally, our results propose a novel *in vitro* model which may prove useful to test potential therapeutic molecules (i.e., anti-inflammatory, antioxidants) for the treatment of DR.

## Data Availability Statement

The raw data supporting the conclusions of this article will be made available by the authors, without undue reservation.

## Author Contributions

CR performed calcium, ROS and TMRM experiments, analyzed data and prepared figures. BB supervised experiments of CK array and rMC-1 activation and participated in manuscript writing. PI performed WB, calcium and ROS experiments. MB performed GSH and ROS experiments. DZ obtained part of funding. AL supported the experiment design with DoE approach. GZ discussed the experiments. FB obtained funding. FG participated in manuscript writing. FC conceived and designed the study, supervised the analyses, and wrote the manuscript. All authors contributed to the article and approved the submitted version.

## Conflict of Interest

AL is founder of Valore Qualità and declares non-competing interests. The remaining authors declare that the research was conducted in the absence of any commercial or financial relationships that could be construed as a potential conflict of interest.

## Publisher’s Note

All claims expressed in this article are solely those of the authors and do not necessarily represent those of their affiliated organizations, or those of the publisher, the editors and the reviewers. Any product that may be evaluated in this article, or claim that may be made by its manufacturer, is not guaranteed or endorsed by the publisher.
